# Measurement of heart rate variability and cognitive abilities based on attachment styles in children with chronic medical conditions

**DOI:** 10.1038/s41598-019-41812-y

**Published:** 2019-03-27

**Authors:** Fahime Ghafarimoghadam, Fateme Dehghani-Arani

**Affiliations:** 1 Department of Psychology, University of Tehran, Dr Kardan Street, Nasr Bridge, Jalal Al Ahmad Street, Chamran Highway, Tehran, Iran; 2 Department of Psychology, University of Tehran, Dr Kardan Street, Nasr Bridge, Jalal Al-Ahmad Street, Chamran Highway, Tehran, Iran

## Abstract

Recognizing the biopsychosocial dimensions of chronic medical conditions in children and preparing them to adapt to medical processes is one of the most significant issues in the field of health psychology. The aim of this study was to measure heart rate variability and cognitive abilities based on attachment style in children with chronic medical conditions. To this end, 45 children aged 12–15 years who had received a diagnosis of a chronic medical disease and were matched with the inclusion/exclusion criteria, were entered the study using available sampling method. These children were assigned to three groups of secure, avoidance and anxiety attachment style based on the Collins and Reid Attachment Scale. These groups had been demographically homogeneous. Then heart rate variability and cognitive abilities were measured. One-way ANOVA results showed a significant difference between the three groups in the heart rate variability and cognitive abilities. Post hoc test showed that children with secure attachment style had higher efficiency in heart rate variability and cognitive abilities. These results indicate that attachment style is one of the factors influencing the health status of children with chronic medical illness. These findings highlight the importance of paying attention to psychological factors, especially attachment and its role in the health status of children with chronic medical conditions.

## Introduction

With the development of theoretical foundations in health psychology, the focus of many studies is on the areas of child health, including the recognition of the psychosocial dimensions of chronic medical conditions. Having chronic disease and its long-term treatment process, along with physical problems, exposes the patient child and his family to high social and psychosocial damages^1,2^ and affects their adjustment in different dimensions^3^. The role of psychological factors in this adjustment is of interest to health psychologists and attachment style is one of these psychological factors^4–7^. It has been specifically addressed in this research.

John Bowlby^8^ considers attachment to be a stable emotional bond that one person forms with another. He emphasizes the necessary of the intimate, warm and continuous relationship between the mother (or her permanent substitute) and the child for the child’s mental health. Recent studies which have focused on attachment in the context of physical illness show the relationship between attachment and medical illness^9^ and the effect of medical illness on attachment characteristics of individuals^10^. David, Black, Coster and Paige^11^ study showed that psychological risk factors such as attachment can predict the growth of chronic disease and its severity in adolescents more than in older ones. And in the studies using psychological interventions in health and medical situations, the significant effects of attachment factor have been confirmed^4,12^.

The stressors of the chronic diseases, challenge individuals and affect psychophysiological responses such as secure responses and cardiovascular functions too. In this regard, heart rate variability (HRV) is an important indicator of anxiety, emotional regulation and health^13^. Studies have shown that anxious people have lower HRV and lower HRV is associated with low psychophysiological coordination, poorer prognosis and increased risk of fatal heart deaths^6^. Psychological interventions can improve the psychophysiological functions of patients and increase their physical and mental health^14^. In connection with the attachment theory, there is also evidence that the secure attachment between the child and the caregiver improves the psychophysiological coordination of the child and reduces painful cardiovascular activity^15^.

On the other hand, the Radleya, Morilakb, Viauc and Campeaud’s study^16^ showed that chronic stress through the activity of the Hypothalamic-Pituitary-Adrenaline (HPA) axis reduces the decision-making process and damages the hippocampal and amygdale areas, leading to decreased activity in the forehead and consequent weakness in cognitive functions. Accordingly, as the main goal of this study, it can be assumed that attachment quality, along with determining the quality of response to stressful conditions and the individual neuronal activities, also affect their cognitive abilities^17^. The cognitive ability of humans has evolved due to the need to solve the ecological problems and to guide complex social environments^18,19^. Stress is one of the important factors leading to cognitive impairment. People who experience high levels of stress often cannot focus on a specific task, and with more cortisol levels, more cognitive impairment occurs^20^. On the other hand, the early experiences during the life contribute to the improvement of the process and the quality of the cognitive abilities. Studies have shown that there is a correlation between childhood injuries such as physical disease, non-primary caregiving and neglect with smaller hippocampal volume and lower cognitive ability during adolescence^21^ and these children are more vulnerable to psychological damage because of the weakness in the ability to solve the problem^22^.

Considering the high vulnerability of children with chronic medical illness during their evolution, attention to attachment style in addition to medical treatment is important in relation to their physical, cognitive and psychological well-being^20,22^. Considering this important issue, the present study, with emphasis on the importance of extending psychological assessments in the field of pediatric health psychology, intends to study the effect of secure, avoidant and anxious attachment styles on cognitive abilities and heart rate variability (HRV) in children with a chronic medical illness. Therefore the main purpose of this study was to identify the differences of HRV and cognitive abilities according to attachment styles in children with chronic medical conditions.

## Results

### HRV subscales

Descriptive Characteristics of the subscales of HRV are presented in Table 1 for the three groups of secure, avoidant and anxious.

**Table 1:** Descriptive indexes for HRV subscales in the secure, avoidant and anxious groups.

HRV subscales	Attachment group	M	SD
Heartbeat	Secure	79.71	4.18
	Avoidant	90.46	4.34
	Anxious	96.12	5.81
	Total	88.77	8.35
Respiration rate	Secure	16.75	0.97
	Avoidant	17.33	1.93
	Anxious	18.61	0.7
	Total	17.56	1.5

In order to evaluate the research hypotheses, the subscales parameters of HRV, including resting heartbeat and respiration rate were evaluated in three groups of children with secure, avoidant and anxious attachment styles. One-way analysis of variance (ANOVA) was used to analyze the data. To this end, the scores of these subscales as dependent variables and group variables (in three levels) as independent variables were entered into the one-way ANOVA.

The ANOVA statistical assumptions for all dependent and independent variables have been implemented. The Kolmogorov-Smirnov test was used to check the distribution of the normality and the Leven test was used to test the consistency of variances.

The results of the ANOVA in Table 2 show that there is a significant difference between the mean of the three groups of secure, avoidant and anxious in the heartbeat (F_2,42_ = 44.48, P = 0.000) and respiration rate (F_2,42_ = 7.86, P = 0.001). Accordingly, at least two groups should have a different mean in the heartbeat and respiration rate. To investigate this, Tukey’s post hoc test was used.

**Table 2:** Results of one-way ANOVA for HRV subscales in the secure, avoidant and anxious groups.

HRV subscales		df	F	Sig.
Heartbeat	Between-group	2	44.48	0.00
	Within-group	42		
	Total	44		
Respiration rate	Between-group	2	7.86	0.00
	Within-group	42		
	Total	44		

P < 0.05.

As shown in Table 3, the heartbeat of subjects with secure attachment is significantly different from those with the avoidant and anxious attachment and it is in fact in the normal range and has a better performance. Concerning the respiratory rate, Tukey’s test showed that there was no significant difference between the secure and the avoidant group, but the difference between the secure group and the anxious group as well as the avoidant group with the anxious group was significant at the level of 0.05.

**Table 3:** Results of post hoc Tuki test for HRV subscales in the secure, avoidant and anxious groups.

HRV subscales	Attacment I	Attacment J	Mean difference	Sig.
Heartbeat	Secure	Avoidant	−10.75	0.00
		Anxious	−16.41	0.00
	Avoidant	Secure	10.75	0.00
		Anxious	−5.66	0.00
	Anxious	Secure	16.41	0.00
		Avoidant	−5.66	0.00
Respiration rate	Secure	Avoidant	−0.58	0.44
		Anxious	−1.86	0.00
	Avoidant	Secure	0.58	0.44
		Anxious	−1.28	0.02
	Anxious	Secure	1.86	0.00
		Avoidant	1.28	0.02

P < 0.05.

### Cognitive abilities Subscales

Further, descriptive statistics derived from the subscales of cognitive abilities of children in the three groups of secure, avoidant and anxious are given in Table 4.

**Table 4:** Descriptive indexes for cognitive abilities’ subscales in the secure, avoidant and anxious groups.

Cognitive abilities’ scales	Cognitive abilities’ subscales	Attachment group	M	SD
Sensory-motor skill	Promptness	Secure	0.48	0.18
		Avoidant	0.36	0.22
		Anxious	0.75	0.24
		Total	0.53	0.26
	Accuracy	Secure	0.39	0.12
		Avoidant	0.7	0.25
		Anxious	0.25	0.15
		Total	0.45	0.26
Visual information processing	Objective sensitivity	Secure	0.58	0.41
		Avoidant	−0.41	0.93
		Anxious	−1.5	1.07
		Total	−0.44	1.19
	Response inhibition	Secure	0.57	0.56
		Avoidant	−2.9	3.44
		Anxious	−3.46	4.41
		Total	1.93	3.65
	Continuous attention	Secure	0.73	0.29
		Avoidant	0.17	0.95
		Anxious	−1.12	1.4
		Total	−0.07	1.25
Working memory	Active memory	Secure	0.71	0.26
		Avoidant	−0.5	1.43
		Anxious	−1.96	1.41
		Total	−0.58	1.59
	Short-term memory	Secure	0.76	0.18
		Avoidant	−0.79	1.61
		Anxious	−0.02	1.32
		Total	−0.01	1.34
	Strategy selection	Secure	1.54	0.78
		Avoidant	0.43	1.88
		Anxious	−1.6	1.16
		Total	0.12	1.86
Effort-error learning	Quick learning	Secure	0.34	0.26
		Avoidant	−0.86	0.89
		Anxious	−1.33	1.09
		Total	−0.61	1.08
	Learning-memory	Secure	0.62	0.27
		Avoidant	−0.53	0.82
		Anxious	−1.55	1.55
		Total	−0.48	1.34

To examine the hypotheses related to cognitive abilities, scales of cognitive abilities including sensory-motor skill (consist of promptness and accuracy subscales), visual information processing (consist of objective sensitivity, response inhibition and continuous attention subscales), working memory (consist of active memory, short-term memory and strategy selection subscales) and effort-error learning (consist of quick learning and learning-memory subscales) were evaluated using one-way ANOVA. In this regard, after ensuring that the ANOVA assumptions were followed, the scores of cognitive abilities subscales were entered as dependent variable and the scores of attachment styles were entered as independent variable.

As the one-way ANOVA results (Table 5) show, in the scale of sensory-motor skills, there is no significant relationship between the mean of promptness subscale and attachment style at the level of 0.05 (F_2,42_ = 0.967, P = 0.388). However, in the case of the accuracy subscale, the difference between secure, avoidant and anxious attachment groups was significant at the level of 0.05 (F_2,42_ = 21.45, P = 0.000). About the visual information processing scale, the difference between secure, avoidant and anxious attachment groups in the subscales of objective sensitivity (F_2,42_ = 42.22, P = 0.000), response inhibition (F_2,42_ = 6.80, P = 0.003) and continuous attention (F_2,42_ = 13.71, P = 0.000) were significant. Active memory (F_2,42_ = 19.58, P = 0.000), short-term memory (F_2,42_ = 6.16, P = 0.004) and strategy selection (F_2,42_ = 20.82, P = 0.000), which are the subscales of the working memory scale, were significantly different in the secure, avoidant and anxious attachment groups. In the subscales related to effort-error learning, the difference between the secure, avoidant and anxious attachment groups in quick learning (F_2,42_ = 16.35, P = 0.000) and learning-memory (F_2,42_ = 16.84, P = 0.000) were significant at 0.05.

**Table 5:** Results of one-way ANOVA for Cognitive abilities’ subscales in the secure, avoidant and anxious groups.

Cognitive abilities’ scales	Cognitive abilities’ subscales		df	F	Sig.
Sensory-motor skill	Promptness	Between-group	2	0.96	0.38
		Within-group	42		
		Total	44		
	Accuracy	Between-group	2	21.45	0.00
		Within-group	42		
		Total	44		
Visual information processing	Objective sensitivity	Between-group	2	42.22	0.00
		Within-group	42		
		Total	44		
	Response inhibition	Between-group	2	6.8	0.00
		Within-group	42		
		Total	44		
	Continuous attention	Between-group	2	13.71	0.00
		Within-group	42		
		Total	44		
Working memory	Active memory	Between-group	2	19.58	0.00
		Within-group	42		
		Total	44		
	Short-term memory	Between-group	2	6.16	0.00
		Within-group	42		
		Total	44		
	Strategy selection	Between-group	2	20.82	0.00
		Within-group	42		
		Total	44		
Effort-error learning	Quick learning	Between-group	2	16.35	0.00
		Within-group	42		
		Total	44		
	Learning-memory	Between-group	2	16.84	0.00
		Within-group	42		
		Total	44		

P < 0.05.

The results of the post hoc test (Table 6) show that the avoidant group has a better function in the accuracy subscale (of the sensory-motor skill scale) than the anxious and secure groups. The anxious group performed weaker than the other two groups in the accuracy dimension.

**Table 6:** Results of post hoc Tuki test for Cognitive abilities’ subscales in the secure, avoidant and anxious groups.

Cognitive abilities’ scales	Cognitive abilities’ subscales	Attachment I	Attachment J	Mean difference	Sig.
Sensory-motor skill	Accuracy	Secure	Avoidant	−0.3	0.00
			Anxious	0.14	0.11
		Avoidant	Secure	0.3	0.00
			Anxious	0.44	0.00
		Anxious	Secure	−0.14	0.11
			Avoidant	−0.44	0.00
Visual information processing	Objective sensitivity	Secure	Avoidant	1	0.00
			Anxious	2.08	0.00
		Avoidant	Secure	−1	0.00
			Anxious	1.08	0.00
		Anxious	Secure	−2	0.00
			Avoidant	−1.08	0.00
	Response inhibition	Secure	Avoidant	3.48	0.01
			Anxious	4.04	0.00
		Avoidant	Secure	−3.48	0.01
			Anxious	0.56	0.88
		Anxious	Secure	−4.04	0.00
			Avoidant	−0.56	0.88
	Continuous attention	Secure	Avoidant	0.56	0.28
			Anxious	1.85	0.00
		Avoidant	Secure	−0.56	0.27
			Anxious	1.29	0.00
		Anxious	Secure	−1.85	0.00
			Avoidant	−1.29	0.00
Working memory	Active memory	Secure	Avoidant	1.21	0.01
			Anxious	2.67	0.00
		Avoidant	Secure	−1.21	0.01
			Anxious	1.45	0.00
		Anxious	Secure	−2.67	0.00
			Avoidant	−1.45	0.00
	Short-term memory	Secure	Avoidant	1.55	0.00
			Anxious	0.78	0.19
		Avoidant	Secure	−1.55	0.00
			Anxious	−0.76	0.2
		Anxious	Secure	−0.78	0.19
			Avoidant	0.76	0.2
	Strategy selection	Secure	Avoidant	1.11	0.07
			Anxious	3.14	0.00
		Avoidant	Secure	−1.11	0.07
			Anxious	2.03	0.00
		Anxious	Secure	−3.14	0.00
			Avoidant	−2.03	0.00
Effort-error learning	Quick learning	Secure	Avoidant	1.21	0.00
			Anxious	1.68	0.00
		Avoidant	Secure	−1.21	0.00
			Anxious	0.46	0.28
		Anxious	Secure	−1.68	0.00
			Avoidant	−0.46	0.28
	Learning-memory	Secure	Avoidant	1.16	0.01
			Anxious	2.17	0.00
		Avoidant	Secure	−1.16	0.01
			Anxious	1.01	0.02
		Anxious	Secure	−2.17	0.00
			Avoidant	−1.01	0.02

P < 0.05.

About the visual information processing scale, there was a significant difference between the secure group and the avoidant and anxious groups in objective sensitivity subscale, and also between the avoidant and the anxious group; The means differences indicate that in this subscale, the secure group had the best function, the avoidant group had a better function than the anxious group and the anxious group had lower efficiency than the other two groups. There is also a significant difference between the secure group and the other two groups in the response inhibition subscale, but there is no significant difference between the avoidant and the anxious groups. Based on the difference in the means, in this subscale, the secure group responded better than the other two groups and the avoidant group was better than the anxious group and the anxious group had the lowest response inhibition. In the continuous attention subscale, there was a significant difference between the secure and anxious groups and also between the anxious and avoidant groups but there was no significant difference between the secure and avoidant groups. Regarding the difference between the mean values, the secure group was more concentrate than the other two groups and the anxious group had the lowest attention.

In working memory scale, the Tukey test showed that the secure group had the highest performance, the avoidant group had better efficiency compared to the anxious group and the anxious group had the lowest performance in the active memory subscale. In short-term memory subscale the secure group had the best performance, the anxious group had a higher performance than the avoidant group and the weakest function was for the avoidant group. And in the strategy selection subscale there is a significant difference between the secure group and the anxious group as well as the avoidant group with the anxious group. The secure group has the highest performance in this subscale and the anxious group has the lowest performance.

Finally about the effort-error learning scale, in both quick learning and learning-memory subscales, the secure group has a significant difference with the other two groups and the other two groups do not have significant differences. The secure group performed better in these two subscales than the other two groups. The avoidant group had a better mean in comparison with anxious group.

## Discussion

In the present study, assessment of the attachment styles, heart rate variability and cognitive abilities in a sample of children with chronic medical condition showed that there is a significant relationship between attachment styles and cardiac and cognitive functions. It can be observed that the children with secure attachment have more normal heartbeat than the avoidant and anxious children; about the respiration rate there were no significant difference between secure and avoidant groups, but the anxious group had a lower efficiency in comparison with the secure and avoidant groups. In cognitive abilities, the results showed that the children with secure attachment have better function in visual information processing, working memory and effort-error learning; but in accuracy subscale of the sensory-motor skill, avoidant group has a better function than the secure and anxious groups.

Along with current study’s finding, dealing with stressors such as illness, individuals with a secure attachment style fill more security, show better cognitive abilities and are more successful in educational, occupational and interpersonal functions^5^. In another study comparing children with secure and unsecure attachment style, secure children showed higher self-esteem in stressful situations, insist on solving the problem, have initiative and perseverance, use more efficient strategies and thus have better physical and mental health^23^. Research done by Morley and Moran^24^ has also confirmed insecurity attachment as a vulnerability factor. In this research, insecure attachment styles are associated with several variables such as anger and hostility, anxiety, depression and behavioral disorders. These indicate the undeniable importance of insecure attachment as a major contributor to biopsychosocial vulnerability in children and adolescents.

Some theoretical studies have identified ways to describe this vulnerability. In this context, based on the opinion of Anderson and Hins^25^ (also in^7,26^) stressors such as disease, activate the attachment style and its associated cognitive-behavioral pattern as well as the neurophysiological responses which can determine the psychophysical health. Therefore, changing the attachment quality will be accompanied by changes in the pattern of individual’s neurophysiological responses and subsequent physical health, as well as changes in cognitive-behavioral patterns and subsequent psychological health. The present study assumes that secure attachment style followed by higher efficiency in HRV and cognitive abilities, because of the better cognitive-behavioral patterns and neuropsychological responses. Anxious children had lower concentration and accuracy but quicker promptness and higher heartbeat than avoidant children, which could be because of their high stress.

On the other side, studies have shown that children’s memory is affected by inconvenient experiences and the attachment style has a mediating role in this relationship. Chae and *et al*.^20^ showed that children with positive mental representations of parents had better function in memory tests. In other words, insecure attachment style has a negative effect on memory performance. These people do not really want to remember the painful events and therefore are more likely to approve false suggestions. It can remind the results of the present study in which secure children had higher performance in memory subscales compared to insecure children. Canterberry and Gillath^27^ assumed that these children are less able to hold the information in their memory for a long time. Maltais, Duchesne, Ratelle and Feng’s study^28^ also found a positive meaningful relationship between learning abilities and success in education with secure attachment. Secure children also had better self-esteem and emotion regulation strategies, and fewer symptoms of anxiety. The present study emphasized the same results in children with chronic medical condition. On the other hand, avoidant children have intensive self-dependency and tend to deny and suppress their negative emotions to deal with high stress; and anxious children have lower concentration, memory and learning performance than children with secure attachment style due to their high stress and negative emotions.

Generally, the present study’s view is that the attachment style affects the patterns of HRV and cognitive abilities in children with medical disease, and these patterns, on the other side, can affect quality of the health conditions. Along with this view, mothers of secure children, in this study, indicated that their children are in harmony with the medical staff and their treatment process goes well, while the mothers of insecure children were always afraid that their child did not have enough co-operation to their treatment, they would have been very upset and even in many cases their illness recurs, resulting in hospitalization. So the present study supports the value and effectiveness of the attachment style in the physical and cognitive functions of children diagnosed with chronic medical conditions. This, along with other studies^1,29^ emphasizes the importance of paying attention to attachment factor in medical treatment plans.

Despite the efforts to control the probable factors, the design was not able to form homogeneous groups with a specific disease, which could have uncontrollable effects on the results of the design. Although attempts were made to reconcile all three groups according to the inclusion/exclusion criteria and matched them according to the demographic information and types of the disease, it is suggested that future studies form more homogeneous groups and assess the role of different factors such as age, sex and type of disease as moderating variables. This study also was a case-control study and the results showed the relationships between attachment, HRV and cognitive factors just among children with medical conditions, rather than comparing with health children. It is suggested for the future studies to compare groups of health and ill children. Consequently, Structural Equation Model would be a potential good method to examine the associations among these multiple factors, which this study could not arrange because of the small sample size limitations. Few participants were among the constraints of this study. It should be noted that, due to limited access to patients, the present study was conducted on a limited sample group, but it is suggested that subsequent studies conduct with more number of participants to increase the validity of the findings. But the present study is just the beginning which can promise the basis for implementation of broader studies that should be done to strongly investigate and discuss the effects of attachment styles on more neuro-psycho-physiological health indexes in children with chronic medical illness.

### Method

The present research was a correlational study with a causal-comparison design. The population was the 12–15 years old children who had received diagnosis of a chronic medical disease. Inclusion criteria were passing at least one month of the medical treatment period, the child’s admission to guidance school education, the parents’ at least primary school education, the basic social and economic income per month. Exclusion criteria were having no history of stroke, addiction, personality and psychotic disorders in parents and children, lack of mental disability in the child, and non-divorce or death of parents. These inclusion and exclusion criteria were checked during an initial interview and also double-checked according to the medical records of the children. Eligible children who were available have been detected and then -after announcement of readiness and agreement- the informed consent form for the participation in the project was signed by the mothers. The form included the researcher’s moral commitment, the engagement commitment of the participant, the participant’s authority to terminate cooperation at each stage of the implementation of the research. Afterwards, the demographic information form and Collins & Reid attachment questionnaire have been completed for the participants. Then, regarding the children’s demographic information and their attachment scores, they were assigned to three demographically matched groups of secure, avoidant and anxious attachment style. The selection procedure has been finished when all three secure, avoidant and anxious groups included 15 children. All three groups were matched in the mean age of children (14.24 with a standard deviation of 0.981), mothers (36.04 with a standard deviation of 4.55) and fathers (40.40 with a standard deviation of 4.46), the average of family income (507.71 $ per month with a standard deviation of 0.85) and disease kind (in each group, 5 children had diabetes, 5 children were suffering from renal failure and 5 children had thalassemia). In the next step, all children in secure, avoidant and anxious groups were evaluated by the Procomp2 tool for measuring HRV and the Cambridge Neuropsychological Test Automated Battery (CANTAB) software for measuring cognitive abilities.

It should be mentioned that this study has been received institutional review board approval from psychology department of University of Tehran and all methods were performed in accordance with the relevant guidelines and regulations.

## Instruments

### Collins and Reid Attachment Questionnaire (1990)

Collins and Reid prepared their questionnaire Based on the Hazan and Shaver’s Adult Attachment Questionnaire which has described three main attachment styles. This self-descriptive 18-item questionnaire measures relationship skills and dominant form of secure, anxious or avoidant attachment style based on three subscales of dependency, proximity and anxiety (each composed of six items). Anxiety subscale measures the extent to which a person is worried about being abandoned or unloved. The proximity subscale measures the extent to which a person is comfortable with closeness and intimacy. The dependency subscale measures the extent to which a person feels he/she can depend on others to be available when needed. As Colins^30^ mentioned, we have defined the subject’s attachment styles based on his/her achieved score in these subscales. Accordingly, secure attachment style identify with a high score on the proximity and dependency and low on the anxiety dimensions; Anxious attachment style identify with a high score on the anxiety, proximity and dependency dimensions; And avoidant attachment style identify with a low score on the proximity, dependency and anxiety dimensions. Scoring method was based on a 5-point Likert scale and a “high” score has been define as being above the midpoint on this scale, and a “low” score as below the midpoint. On this view, individuals who score at the midpoint should be excluded from the sample. The test-retest reliability of this questionnaire was reported more than 80%^31,32^. The results of Pakdaman research^33^ also showed that the validity of the Persian version of this questionnaire was 76%. In the current study the coefficient of Cronbach’s Alpha was 0.75.

### Electrocardiogram (ECG)

ECG was used to measure HRV. To assess the heartbeat and respiration rate, this tool detects electric shocks and records waves across the skin. An ECG heartbeat includes a P wave, a T wave, a U wave and a QRS set. Each of these waves and the distances between them are related to different parts of the cardiac function and can be used to evaluate the health of the heart. The wave R represents the contraction of the ventricles (heartbeat). R-R is the heartbeat interval, or the normal to normal (NN) distance, and represents the distance between heartbeats. The device used to measure HRV in this study was ProComp 2, which shows heartbeat and respiration rate which was measured in resting mode. To do this, the front of the instrument’s wire was connected to the participant’s left hand and the end of the wire was inserted into the device’s B channel. The next wire was added to the H channel of the device to measure the heartbeat of the chest.

### Cambridge Neuropsychological Test Automated Battery (CANTAB)

The neuropsychological tests which have been presented in this tool were originally prepared in 1980 by Sahakian, Robbins and Freez at Cambridge University. This tool has 7 scales which measure cognitive abilities such as memory and learning, executive function and active memory, visual memory, attention and reaction time, semantic and verbal memory, decision-making and response control, and social cognition. These scales included 24 subscales. The results of the aforementioned abilities and subscales refer automatically to determined ranges of norms matched according to age and gender, and standard scores automatically provide by the CANTAB^34^. The primary objective of this software was to evaluate the cognitive decline function in elderly people with dementia; but in the following years, it has been used for evaluation of the neuropsychological functions in clinical populations and different age groups with different demographic characteristics^35^. The results of the studies showed a test-retest correlation of 56% to 86% for the subscales^35,36^. The coefficient of Cronbach’s Alpha for the current study’s sample was 0.87.
